# A 3D analysis revealed complexe mitochondria morphologies in porcine cumulus cells

**DOI:** 10.1038/s41598-022-19723-2

**Published:** 2022-09-13

**Authors:** Amel Lounas, Ariane Lebrun, Isabelle Laflamme, Nathalie Vernoux, Julie Savage, Marie-Ève Tremblay, Marc Germain, François J. Richard

**Affiliations:** 1 Centre de Recherche en Reproduction, Développement et Santé Intergénérationnelle (CRDSI), Département des Sciences Animales, Faculté des Sciences de L’agriculture et de L’alimentation,, Université Laval, Québec, QC G1V 0A6 Canada; 2 Département de Biologie Médicale, Université du Québec À Trois-Rivières, Québec, G8Z 4M3 Canada; 3 Centre de Recherche du CHU de Québec-Université Laval, Axe Neurosciences, Département de Médecine Moléculaire, Université Laval, Québec, QC G1V 4G2 Canada; 4 Division of Medical Sciences, University of Victoria, Victoria, BC V8W 2Y2 Canada

**Keywords:** Cell biology, Physiology, Reproductive biology

## Abstract

In the ovarian follicle, a bilateral cell-to-cell communication exists between the female germ cell and the cumulus cells which surround the oocyte. This communication allows the transit of small size molecules known to impact oocyte developmental competence. Pyruvate derivatives produced by mitochondria, are one of these transferred molecules. Interestingly, mitochondria may adopt a variety of morphologies to regulate their functions. In this study, we described mitochondrial morphologies in porcine cumulus cells. Active mitochondria were stained with TMRM (Tetramethylrhodamine, Methyl Ester, Perchlorate) and observed with 2D confocal microscopy showing mitochondria of different morphologies such as short, intermediate, long, and very long. The number of mitochondria of each phenotype was quantified in cells and the results showed that most cells contained elongated mitochondria. Scanning electron microscopy (SEM) analysis confirmed at nanoscale resolution the different mitochondrial morphologies including round, short, intermediate, and long. Interestingly, 3D visualisation by focused ion-beam scanning electron microscopy (FIB-SEM) revealed different complex mitochondrial morphologies including connected clusters of different sizes, branched mitochondria, as well as individual mitochondria. Since mitochondrial dynamics is a key regulator of function, the description of the mitochondrial network organisation will allow to further study mitochondrial dynamics in cumulus cells in response to various conditions such as in vitro maturation.

## Introduction

The female germ cell nests in an ovarian follicle in which the oocyte is surrounded by both of cumulus cells, granulosa cells, a basal lamina and the theca cells. The growth of the ovarian follicle and the development of the oocyte are closely related and depend on the relation of the oocyte with adjacent somatic cells. During folliculogenesis, a bilateral cell-to-cell communication is established between the oocyte and cumulus cells that is known to impact the oocyte growth and development. These interactions may use direct transfer of molecules between cells by gap junction communication or by transzonal projections, and may use paracrine factors through receptors or membrane interactions^1^. The transfer of pyruvate to the oocyte through gap junctions was shown to be very beneficial for oocyte maturation and oocyte quality^2^. Interestingly, in cumulus cells, pyruvate derivatives produced by mitochondria can transit through gap junctions^1,3^.

Mitochondria are key intracellular organelles that produce most of the ATP needed by the cell to maintain homeostasis and meet metabolic needs. However, mitochondria do much more than simply participating in the energy metabolism of the cell. In addition to producing several important metabolites and regulating various signaling pathways, they play major roles in different physiological processes including stem cell maintenance, cell differentiation, and apoptosis^4–7^. In recent years, several studies demonstrated that the various mitochondrial shapes and sizes correspond to different functions^8^. The morphological changes of mitochondria which are controlled by the continuous fusion and fission processes, provide important information about the physiological state of mitochondria as well as of the cell^5,9,10^. For example, fragmented mitochondria are characterised by low oxidative capacity while elongated forms have higher ATP and oxidative phosphorylation levels^11^. In addition, mitochondrial fragmentation is associated with the remodeling of cristae to facilitate cytochrome c release during apoptosis in response to cellular stress^12,13^. Starvation and nutrient restriction increase mitochondrial length and induce tighter cristae to maintain ATP synthesis and cell survival^14^. Compromised mitochondrial dynamics could therefore affect mitochondrial functions as observed in various disorders including reproductive pathologies such as ovarian dysfunction^15^.

In active steroidogenic cells such as cumulus cells, mitochondria are also involved in steroid biosynthesis. In these cells type, the cholesterol is transported by the Star protein (steroidogenic acute regulatory protein) from the outer to the inner mitochondrial membrane and is converted into pregnenolone^16^. In the oocyte, mitochondria are involved in energy production, as well as in the transmission of the maternal mitochondrial genome to the next generation^17^. Furthermore, both of the mitochondrial number and the mitochondrial copy genome are amplified during oogenesis and oocyte growth to support the elevated energy requirements of meiotic resumption and oocyte development^18,19^. In premature porcine oocytes, a positive correlation was observed between mitochondrial DNA (mtDNA) copy number in the cumulus cells and that in oocytes reflecting the contribution of cumulus cells mitochondria in oocyte developpement^20^. Interestingly, it was demonstrated that mitochondrial dysfunction in cumulus cells affects oocyte quality^21,22^.

In Cumulus-Oocyte Complex (COC), cumulus cells play an important role in oocyte maturation^3^. It is also well known that the metabolic status of cumulus cells involving mitochondria is a critical factor for the oocyte development competence acquisition^23^. In this context, understanding the mitochondrial morphological changes in cumulus cells is of great interest in cellular physiology to further understand its role. Since mitochondrial dynamics is a key regulator of mitochondrial function, we described the morphology of mitochondria using confocal and scanning electron microscopy in pig cumulus cells immediately after removal from the follicular environment. Furthermore, we provided a three-dimensional reconstruction of mitochondrial morphology, and we measured their volume as well as other parameters. Our observations support the presence of different complex mitochondrial morphologies in cumulus cells. The results provide fundamental information for future studies on the effects of in vitro maturation *(IVM)* on mitochondria in cumulus cells.

## Results

### Confocal microscopy analysis of mitochondrial phenotypes in cumulus cells

**Figure 1:**
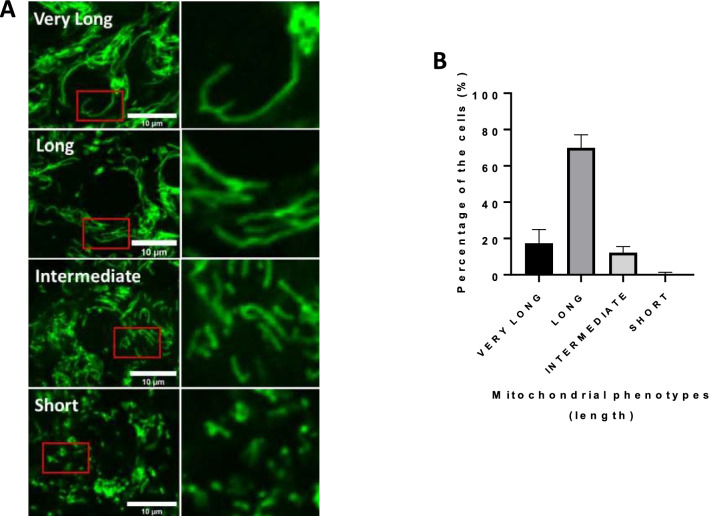
Staining of active mitochondria. (**A**) Images of cumulus cells stained with TMRM and observed live with confocal microscopy at 63X (n = 3). Different mitochondrial network structures in cumulus cells were observed. (**B**) Mitochondrial network phenotype evaluation in each cumulus cell (n = 3, mean ± SD).

**Figure 2:**
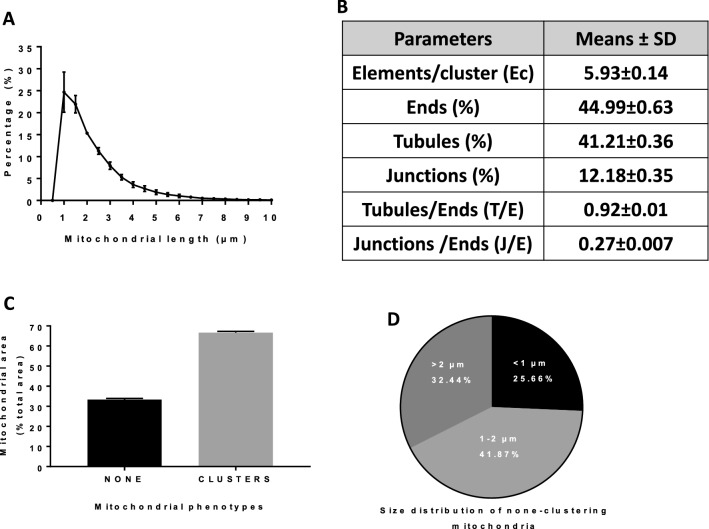
Momito analysis of mitochondrial morphologies in cumulus cells using confocal images. (**A**) Quantification of mitochondrial length distribution. (**B**) Parameters of mitochondrial network. (**C**) Area percentage covered by each mitochondrial phenotype. (**D**) Size distribution of none-clustering mitochondria. (n = 3, mean ± SD).

To determine the morphology of mitochondria within cumulus cells, we stained active mitochondria with the TMRM (Tetramethylrhodamine, Methyl Ester, Perchlorate) dye which is sensitive to mitochondrial membrane potential, and we observed cells using confocal microscopy. Images of stained organelles revealed different mitochondrial morphologies in cumulus cells varying between short, intermediate, long, and very long (Fig. 1A). To evaluate the mitochondrial phenotype, each cumulus cell (482 cells from three replicates) was manually quantified as having short, intermediate, or long mitochondria. We found that only 0.5 and 12% of the cells had short and intermediate mitochondria, respectively. Most cells had long and very long mitochondria, corresponding respectively to 70% and 17% of the cells (Fig. 1B). To complement these observations, we used the Momito algorithm measuring the mitochondrial length which varied between 0.5 and 7 µm with 40% of mitochondria longer than 2 µm (Fig. 2A). Thus, these two analyses from TMRM staining revealed that, in cumulus cells, mitochondria are mostly long.

Additional mitochondrial parameters were measured with the Momito algorithm^24^, including the percentage of ends, tubules, and junctions as they illustrate the complexity and the connectivity of the organelle network. The connected mitochondria with more than seven elements (ends, tubules, and junctions) were identified as clustered forms. We noted a mean of 5.93 ± 0.14 elements in each mitochondrial cluster supporting the prevalence of complex and branched clustered forms, and we calculated a connectivity of 0.27 suggesting the presence of connected components (Fig. 2B). To evaluate the size of mitochondrial clusters in cumulus cells, we calculated the area covered by each mitochondrial phenotype. We observed that 66.64% of the total mitochondrial area was covered by clustered forms suggesting that they occupied the largest area in the mitochondrial network (Fig. 2C). The size distribution of non-clustered mitochondria which covered 33.35% of the total mitochondrial area was also analysed. We observed that isolated organelles with more than 2 µm in length covered 32.44% of the total isolated mitochondrial area. Based on Momito results, we supported that the mitochondria from cumulus cells are mainly connected (Fig. 2C) and long (Fig. 2D). In addition, the disruption of mitochondrial membrane potential by rotenone treatment (an inhibitor of the mitochondrial respiratory chain complex I of the electron transport chain) for two hours revealed a significant reduction in mitochondrial length quantified as a shift in mitochondrial length distribution (Fig. S1A,B in supplemental data). The calculated Earthmover distance (EMD) values which are a relative distance between two distributions^25^ showed a significant difference between the control and the rotenone treatments (Fig. S1C in supplemental data). In addition, mitochondrial connectivity was also decreased in cumulus cells treated with rotenone compared to control (Fig. S1D in supplemental data). The results provide evidence that mitochondria of cumulus cells are sensitive and active.

### Ultrastructural analysis of mitochondrial morphology in cumulus cells

**Figure 3:**
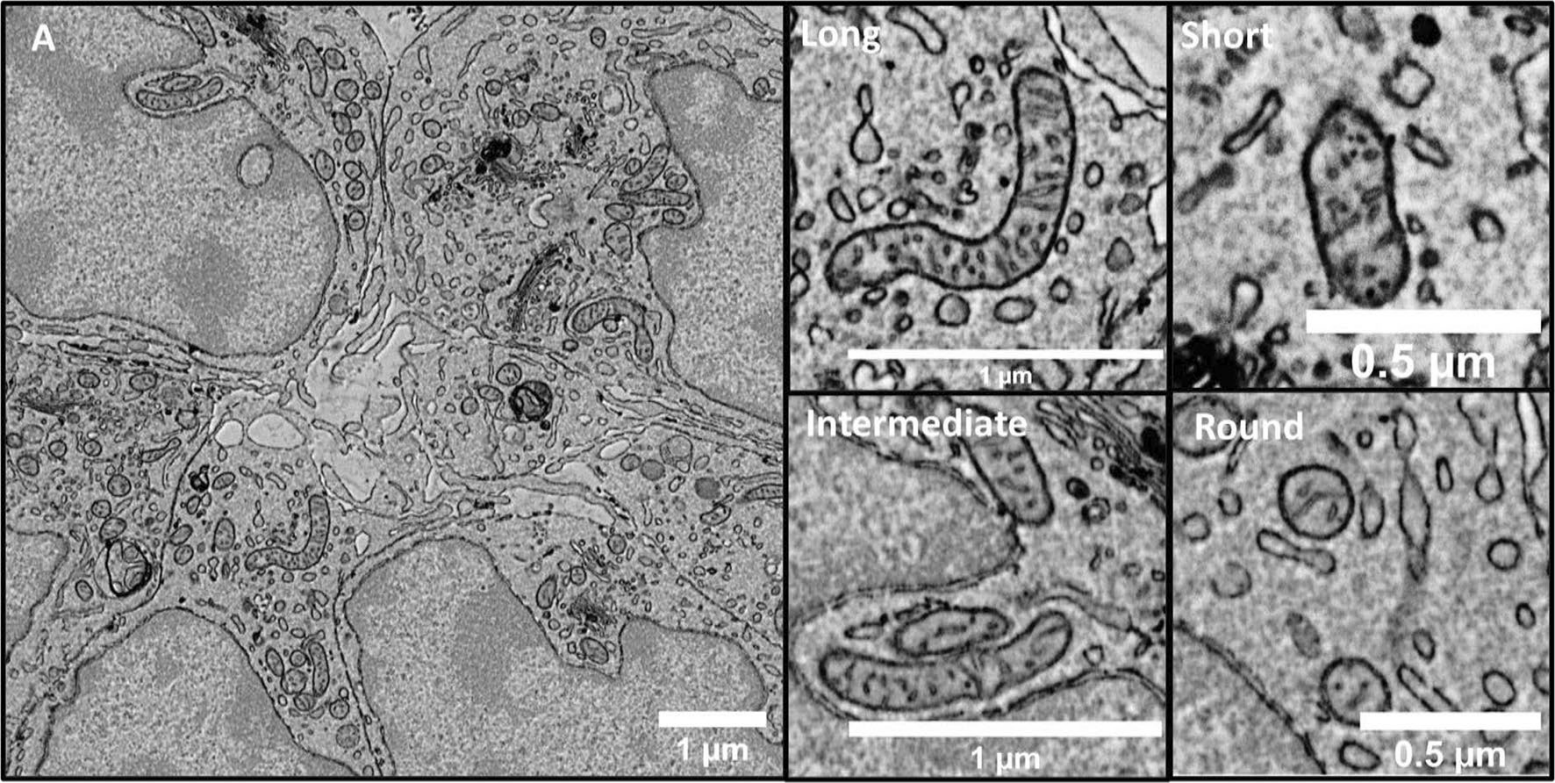
Scanning electron microscopy micrographs showing whole cells (**A**) and different mitochondrial morphologies in cumulus cells (long, intermediate, short, round).

**Figure 4:**
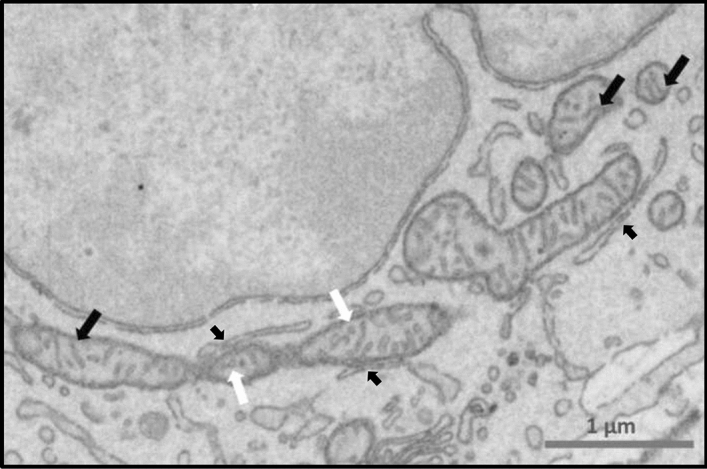
Cumulus cell scanning electron microscopy micrograph showing: examples of mitochondria with lamellar cristae (black arrow) and tubular cristae (white arrow). Mitochondria in the top right of the image show a developed lamellar crista connected to the inner mitochondrial membrane. Several close contacts (mitochondria-associated membranes, MAM) between mitochondria and endoplasmic reticulum in cumulus cells (small black arrows).

To further describe mitochondrial shapes and sizes in cumulus cells, Scanning electron microscopy (SEM) analysis was first performed. The general morphology of cumulus cells was well defined with a large nucleus and a small amount of cytoplasm containing different organelles such as mitochondria, endoplasmic reticulum (ER), Golgi, and small vesicles (Fig. 3A). Different structural appearances and dimensions of mitochondria were observed including round, short, intermediate, and long (Fig. 3). Detailed ultrastructural analysis at nanoscale resolution showed that mitochondrial cristae were well developed, and we observed both lamellar and tubular cristae (Fig. 4). Unlike tubular cristae, lamellar cristae were attached to inner mitochondrial membranes, as previously described^26^.

Relative to cell size, mitochondria occupied 3.08 ± 1.44% of all cell surface (Table S1 in supplemental data). They displayed a typical distribution within the cytoplasm: in the vicinity of the nucleus and the ER. A lot of mitochondria-ER contact sites (MERCS) were observed in cumulus cells suggesting an active physiological communication between these two organelles (Fig. 4). In addition, several inter-mitochondrial junctions were observed, showing a specific cristae alignment as previously described, which further supports an inter-mitochondrial communication^27^ (Fig. S2 in supplemental data).

**Figure 5:**
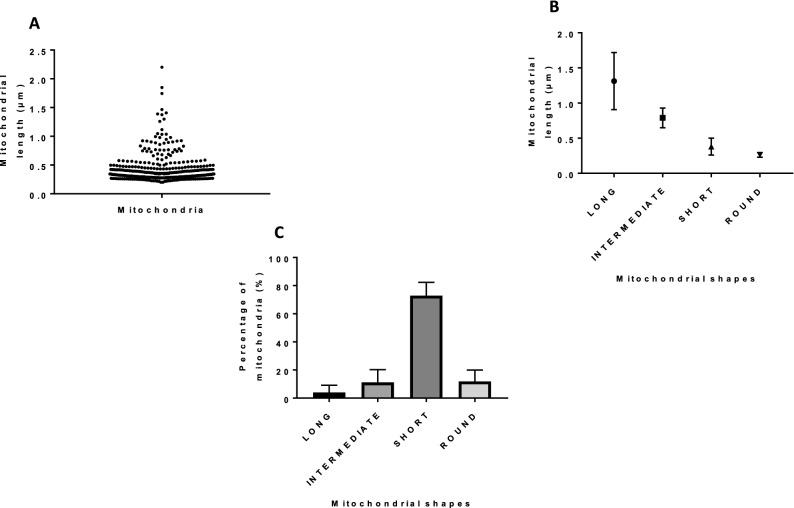
Fiji analysis of scanning electron microscopy micrographs. (**A**) Length distribution of all measured mitochondria. (**B**) Classification of mitochondrial shapes from 16 cells. (**C**) Percentage of each mitochondrial shape (n = 3, mean ± SD).

Further analysis of the SEM images with the Fiji software allowed us to properly analyse 326 organelles length (from 16 cells) which varied between 0.2 and 2.2 µm (Fig. 5A). Based on these measurements and mitochondrial shapes, we developed four different organelle categories: round, short, intermediate, and long (Table S2 in supplemental data), supporting the presence of varied mitochondrial morphologies and sizes in cumulus cells (Fig. 5B). Both confocal and SEM imaging suggested that mitochondria from cumulus cells had diversified forms with different sizes. Although SEM images showed a larger proportion of short organelles (Fig. 5C) contrarily to the abundance of the elongated phenotype by confocal analysis, this discrepancy could be explained by the limitation of SEM to a 2-dimensional plane with a limited depth of 10 nm which led us to move on to 3D analyses.

### Three-dimensional reconstruction of the mitochondrial network in cumulus cells

**Figure 6:**
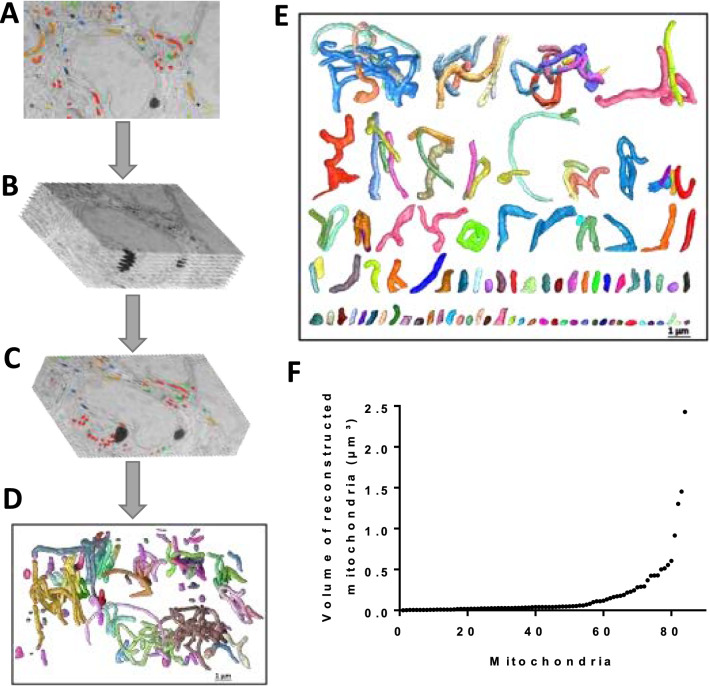
Dragonfly reconstruction of mitochondria in five cumulus cells using FIB-SEM micrographs. (**A**) 2D-electron micrograph of segmented cells. (**B**,**C**) Serial images of FIB-SEM used for 3-D reconstruction. (**D**) Three-dimensional organisation of all mitochondrial networks in 5 cumulus cells. (**E**) Different reconstructed mitochondrial structures ranked top to bottom from the largest to the smallest volume (**F**) mitochondrial volume quantification.

To resolve the conflicting observations obtained by confocal and SEM microscopy analysis and to complete our description of mitochondrial morphology in cumulus cells, we reconstructed the 3D ultrastructure of all mitochondria from five cumulus cells using serial images (5 nm in XY and 10 nm in Z) obtained by FIB-SEM microscopy (Fig. 6A–C). Three-dimensional reconstruction of individual mitochondria and their classification from the largest to the smallest volume revealed complex structures with different forms and sizes as well as connected clusters (Fig. 6D,E). The total volume of all mitochondrial fractions was 14.44 µm^3^, the largest cluster volume was 2.4 µm^3^ while the smallest individual mitochondria measured 0.0015 µm^3^ (Fig. 6F), a volume 1500 times smaller than the largest cluster.

When examining all the reconstructed mitochondria in the network (Fig. 6E), we found that larger clusters contained more than twenty elements of ends, tubules, and junctions. In contrast, individual branched mitochondria contained fewer than ten elements; and depending on whether they had junction points or not, they could have as little as three elements: two ends and one tubule (isolated mitochondria). The largest reconstructed cluster contained six interconnected components which had several branches > 1 µm in length. In addition, one of the six components showed a hyper-connected organisation of numerous attached mitochondria (Fig. S3 in supplemental data).

**Figure 7:**
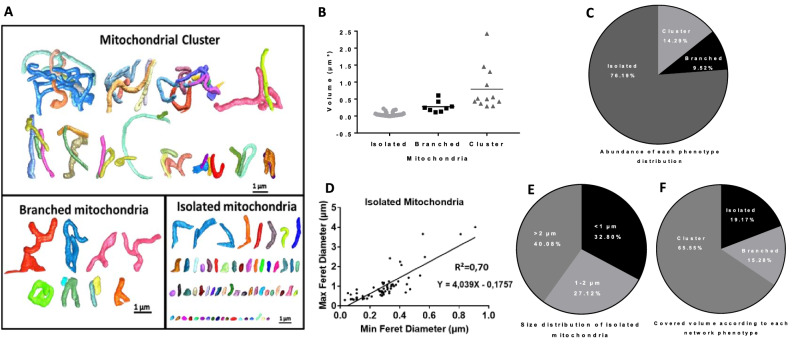
Mitochondrial parameters analysed with Dragonfly. (**A**) Network classification **in**to clustered, branched, and isolated forms. (**B**) Volume distribution of each mitochondrial category. (**C**) Abundance of each mitochondrial phenotype. (**D**) Relationship between Min ferret and Max ferret in isolated mitochondria. (**E**) Size distribution of isolated mitochondria. (**F**) Covered volume according to each mitochondrial phenotype.

Then, we classified mitochondria based on their structure into clustered, branched, or isolated forms (Fig. 7A) and we determined the volume distribution of each category as well as their prevalence. The smallest cluster was 0.28 µm^3^ in volume, branched forms volume varied between 0.11 and 0.6 µm^3^, while the volume of the largest isolated organelles was 0.21 µm^3^ (Fig. 7B). However, isolated forms were more abundant (76.19%) than clusters (14.29%) and branched (9.52%) mitochondria (Fig. 7C). The minimum and the maximum ferret diameters of isolated mitochondria were measured with the Dragonfly software to assess their roundness. Their length varied between 0.3 and 4.1 µm while their maximum width was 0.9 µm (Fig. 7D), which revealed that isolated organelles were not round. In addition, the isolated mitochondrial population longer than 2 µm and 1–2 µm in length covered 40.08% and 27.12% of the total isolated mitochondria volume respectively. These observations suggested that most of the isolated mitochondria measured over 1 µm in length (Fig. 7E).

The number of cristae in each mitochondrial category was also evaluated as well as their diameter to determine whether clustered forms had more developed cristae than isolated mitochondria. There was no significant difference in the number of cristae between the different mitochondrial forms (Fig. S4 in supplemental data). Interestingly, the analysis of the covered volume according to each mitochondrial phenotype revealed that clustered forms covered more than 65% of all mitochondrial volume (Fig. 7F). These results suggested that although clustered forms were not abundant (14.29%), they occupied the largest volume compared to the other phenotypes.

## Discussion

This work described mitochondrial morphology organisation in porcine cumulus cells immediately after removal from the follicular environment using different methodological strategies. We first used confocal microscopy analysis to examine different mitochondrial morphologies (long, intermediate, and short). Measurement of various parameters with the Momito algorithm determined that mitochondria are mainly long and connected in cumulus cells. The 3D visualisation of mitochondria by FIB-SEM revealed that mitochondria are not only long, but also form a network with clusters and branched structures.

Similar to our results, previous confocal microscopy observations of mitochondria from various cell types revealed different sizes (long, short, round)^28–30^. To segment mitochondria and measure their network parameters in confocal images, several automated approaches were used. These methods provided information about mitochondrial morphology and dimensions, but most of them underestimated the length^31,32^. In the present study, we used the Momito algorithm, which is a probabilistic approach allowing to measure mitochondrial length distribution and connectivity^24^. This algorithm is a powerful tool to study mitochondrial network phenotypes in cells without any prior information about organelle shapes. With this tool, we obtained a global view of the mitochondrial population morphology in cumulus cells, and we measured both their length and connectivity. A reduction in mitochondrial length as well as in mitochondrial connectivity was also reported after rotenone treatment.

Electron microscopy has long been used as a primary tool to reveal at nanoscale resolution the ultrastructure of cells and organelles in two dimensions. Comparable to our results, different mitochondrial morphologies were reported in various cell types from numerous species^33–35^. In mouse cumulus cells, previous studies revealed that mitochondria appeared tubular with transverse cristae^21,36^. Moreover, elongated mitochondria were observed in human cumulus cell^37^. All these descriptions were based on observations in two dimensions by electron microscopy. However, it has been demonstrated that depending on the mitochondrial distribution in cells, the organelle could be cut in different orientations (transversely, obliquely or longitudinally) resulting in varied mitochondrial shapes (elongated, oval or circular) when observed in two dimensions^35^. Therefore, 3D reconstruction of mitochondria is essential for a proper description of the mitochondrial network. Effectively, 3D characterisation of mitochondria in cumulus cells first confirmed the presence of the elongated mitochondrial phenotype observed by both confocal and FIB-SEM microscopy. A mitochondrial network with clustered structures was also highlighted and analysed by the Momito algorithm and validated by 3D analysis.

Recently, several studies focused on the 3D reconstruction of mitochondria from various cell types^8,38,39^. Through this new approach, a quantitative 3D analysis was developed to assess numerous mitochondrial parameters. Comparable to our 3D findings, mitochondria from healthy human skeletal muscles displayed a large heterogeneity of morphology and volume (small, large, branched, and complex mitochondria) within the same cell^8^. Thus, the largest mitochondria from human skeletal muscle measured 0.68 µm^3^ while those from mouse cells measured 4.19 µm^3^. In addition, skeletal muscle cells of patients with mitochondrial diseases contained more simple fragmented mitochondria compared to cells from healthy individuals which contained a large number of branched mitochondria^8^. This morphological feature of organelle network was associated with mitochondrial stress in human muscle cells^8^. Furthermore, 3 D reconstruction of mitochondria from cardiac muscle cells showed that the network organisation as well as individual mitochondrion shapes and sizes correlated with cellular energy demands^40^. In mammalian cells, it was reported that 3D reconstructed individual mitochondria communicated with each other by nanotunnels allowing the formation of dynamic mitochondrial networks^41^. Moreover, the 3D organisation of mitochondria in neuronal cells showed the predominance of tubular structures in axons and dendrites suggesting their crucial role in the maintenance of cellular energy homeostasis^42^.

Based on these previous observations, the complexity of the mitochondrial network organisation in cumulus cells is expected to support important physiological functions. For example, the prevalence of the elongated phenotype in cumulus cells could reflect the high energy requirement to support the different physiological processes of the oocyte such as meiosis, maturation, and fertilization. In addition, increased interconnected and branched components in the mitochondrial network suggested a high level of signal transfer to support functions such as energy production^27,43^. Moreover, inter-mitochondrial junctions with specific cristae alignments were associated with the regulation of mitochondrial communication as well as electrochemical gradient transfer for energy synthesis^44^. Furthermore, the numerous contacts between mitochondria and ER (mitochondria-associated ER membranes) in cumulus cells suggested a high ER-mitochondrial calcium transfer^45^ which may be part of a physiological process involved in oocyte maturation^46,47^. Mitochondria-associated ER membranes are also implicated in lipid synthesis^48^, and lipid metabolism in cumulus cells contributes to oocyte metabolism homeostasis and can affect meiotic resumption^49^. The mitochondrial morphological features observed in this study are consistent with the role of cumulus cells in the regulation of oocyte physiology and events such as meiosis and maturation.

In conclusion, the fine characterisation of mitochondrial morphology described in this paper showed a complex organisation of the organelle network in cumulus cells right after removal from the follicular environment. Although, more work remains to be done to further understand the link between mitochondrial structure and function in cumulus cells. Our results establish a clear picture of the mitochondrial network that could be used to further study the regulation of mitochondrial responses during *IVM*. Thus, the modulation of mitochondrial response in cumulus cells could be seen as a novel strategy to improve oocyte developmental competence.

## Experimental procedures

### Chemicals

Unless otherwise stated, all chemicals were purchased from Sigma Chemical Company (St. Louis, MO, USA).

### Recovery of cumulus-oocyte complexes

Pig ovaries were collected from pre-pubertal animals as previously described^50^. In brief, ovaries were recovered from a local slaughterhouse, placed in saline solution (0.9% NaCl) including antibiotics and antimycotics (100,000 IU/L penicillin G, 100 mg/L streptomycin, 250 μg/L amphotericin B) and maintained at 37 °C. Once in the laboratory, ovaries were rinsed in saline solution and antral follicles (3–6 mm) were punctured by an 18-gauge needle attached to a 10-mL syringe to aspirate follicular cells in follicular fluid. Cumulus-oocyte complexes (COC) were recovered and washed with HEPES-buffered Tyrode medium containing 0.01% (w/v) polyvinyl alcohol (PVA-HEPES)^51^ and selected according to criteria described previously^52^. The cells were used immediately or fixed for future use.

### Staining of active mitochondria

Active mitochondria from 30 fresh COC were labeled with TMRM (Tetramethylrhodamine, Methyl Ester, Perchlorate, ThermoFisher Scientific, Waltham, MA, USA) at a final concentration of 150 nM for 30 min at 37.5 °C^53^. This dye is very sensitive to mitochondrial membrane potential and accumulates in active mitochondria from healthy cells. The COC were then washed 3 times with PBS and mounted on glass slides using Grace Bio-Labs 200 SecureSeal imaging spacers. Cumulus cells were imaged live on a confocal live–cell LSM700 microscope and images were taken with ZEN capture at 63X magnification.

### Scanning electron microscopy

The COC were prepared according to an adapted protocol as described previously with some modifications^54^. Immediately after recovery, two hundred fresh COC were fixed in 3.5% acrolein solution for 30 min at room temperature and washed twice with PBS. Cells were pelleted by a quick centrifugation and mixed with 4% agarose to make a solid mold. Thin sections (50 μm) were then obtained using a vibratome (Leica VT1000S, Leica Biosystems, Concord, ON, Canada). They were post fixed 1 h in a solution containing 3% potassium ferrocyanide combined with an equal volume of 4% aqueous osmium tetroxide (Electron Microscopy Sciences; EMS) and were washed five times with double-distilled water (ddH_2_O) for 3 min each. Tissues were then placed in filtered (0.22 µm Millipore) thiocarbohydrazide solution (TCH) for 20 min at room temperature. They were then rinsed again four times with ddH_2_O, and post fixed a second time in 2% osmium tetroxide for 30 min at room temperature. The sections were then dehydrated with sequential alcohol baths and propylene oxide, transferred to Durcupan resin (EMS) between two ACLAR sheets (EMS) and placed in the oven at 55 °C for 3 days. Ultrathin sections (70 nm) of the region of interest of the 50 μm sections were prepared with a Leica UC7 ultramicrotome. Images of cumulus cells (1 nm resolution) were acquired using a Zeiss Crossbeam 540 scanning electron microscope.

### Sample preparation for focused-ion beam scanning electron microscopy (FIB-SEM)

Focused-ion beam Scanning electron microscopy (FIB-SEM) was performed as previously described^55^. Briefly, the prepared COC tissues were trimmed into a trapezoidal prism using a glass knife. A second glass knife was used to polish the surface of the tissues and the trimmed block was removed using a jeweler’s blade and mounted on an aluminum stub (EMS) using conductive carbon paint (EMS). The sample was sputtered with 30 nm of platinum using a sputter coater (Zeiss) and loaded into a Zeiss Crossbeam 540 FIB-SEM. Once in the FIB-SEM, the cumulus cell layer was identified for iterative FIB-milling and SEM imaging. The ATLAS Engine 5 software (Fibics) was used to automate the steps involved in FIB-SEM data collection. Images of cumulus cells were acquired with 5 nm pixels in the lateral dimensions using the ESB and SE2 detectors with the SEM voltage set at 1.4 kV and current of 1.2 nA. The FIB milling steps of 10 nm were accomplished using a milling voltage of 30 kV and current of 1.5 nA. Volumes of 125 μm^3^ were captured over 18- to 24-h imaging sessions. After acquisition, image stacks were finely aligned using the ATLAS software. We collected 300 serial images with 10 nm in z-direction getting 3 µm of total imaging depth.

### Image analysis

To conduct a full investigation of the mitochondrial network, images were used for both qualitative and quantitative analysis. Confocal images of the mitochondrial network were analysed with the Momito algorithm to measure mitochondrial length distribution in cumulus cells as previously described^24^. First, confocal images (n = 30 COC) were converted to binary images, then several clusters were obtained from separated mitochondrial networks which were analysed by the structure interpreter. Information about the network structure of each cluster was used to generate the whole cell distribution probability. On the other hand, the Fiji software was used to measure mitochondrial length from the scanning electron microscopy images of 16 cumulus cells from the same COC. In addition, three-dimensional reconstruction of mitochondrial network was done with the Dragonfly software (Ver. 2020.1; Object Research Systems, Montreal QC). Mitochondria from five cumulus cells were segmented using the U-Net convolutional neural network^56^ within the deep-learning tool in Dragonfly. To train our model, 15 fully labeled (mitochondria and all other cell components) areas with different sizes were taken from 15 FIB-SEM images. The total number of pixels in these 15 areas was 16,822,622, of which 1,092,273 were from mitochondria and 15,730,349 from other components. When the model was well trained (dice score of 99.2%), it was applied to segment all mitochondria from 300 slices through a 3-µm depth followed by a full manual correction. Finally, the watershed mapping approach was used to segment individual mitochondria in clusters.

## Supplementary Information

Supplementary Information.

## Data Availability

All data generated during this study are included in this published article.
