# Supplementary material for: A 3D analysis revealed complexe mitochondria morphologies in porcine cumulus cells
Source: Sci Rep. 2022 Sep 13;12:15403. doi: 10.1038/s41598-022-19723-2 (PMC9470746; doi:10.1038/s41598-022-19723-2)
Supplement: Supplementary file 1 — Supplementary Information. [file 41598_2022_19723_MOESM1_ESM.pdf]

Supplemental data

A 3D analysis revealed complexe mitochondria morphologies in porcine cumulus cells

Amel Lounas[1], Ariane Lebrun[1], Isabelle Laflamme[1], Nathalie Vernoux[3], Julie Savage[3], Marie-Ève Tremblay[3,4], Marc Germain[2]& François J. Richard[1]

[1]Centre de recherche en reproduction, développement et santé intergénérationnelle (CRDSI), Département des sciences animales, Faculté des Sciences de l'agriculture et de l'alimentation, Université Laval, Québec, Québec, G1V 0A6, Canada.

[2]Département de biologie médicale, Université du Québec à Trois-Rivières, Québec, G8Z 4M3, Canada.

[3]Centre de recherche du CHU de Québec-Université Laval, Axe Neurosciences, Département de médecine moléculaire, Université Laval, Québec, Québec, G1V 4G2 Canada

[4]Division of Medical Sciences, University of Victoria, Victoria BC, V8W 2Y2 Canada

Table 1: Area of cumulus cells, nuclei, cytosol and mitochondria. The area percentages were calculated based on cell surface. The results are presented as means ± sd. (n = 16 cumulus cells)

Parameter	Cell number	Whole Cells	Nuclei	Cytosol	Mitochondria
**Area (µm)**	16	58.74 ± 16.38	23.85 ± 8.79	33.16 ± 10.94	1.72 ± 0.81
**Area percentage (%)**	-	100	40.29 ± 13.42	56.62 ± 12.46	3.08 ± 1.44

Table 2: The length of different mitochondrial categories observed by SEM microscopy.

Parameter	Long	Intermediate	Short	Round
**Length category**	>1µm	0.7-1µm	0.3-0.7µm	<0.3µm

Materials and Methods

Disruption of mitochondrial membrane potential with Rotenone

Experimental Procedure: Immediately after recovery, 10 COC (per replica, n=3) were incubated in the presence of rotenone (5 µM) for 2 hours at 37.5 °C and active mitochondria were stained with TMRM (150 nM) for 30 min at 37.5 °C before imaging. Control COC were not exposed to rotenone but were also stained with TMRM before imaging. The COC were then washed 3 times with PBS and mounted on glass slides using Grace Bio-Labs 200 SecureSeal imaging spacers. Cumulus cells were imaged live on a confocal live–cell LSM700 microscope and images were taken with ZEN capture at 63X magnification.

Images analysis: Confocal images of the mitochondrial network were analysed with the Momito algorithm to measure mitochondrial length distribution in cumulus cells from each experimental condition (Ouellet et all, 2017). The Earthmover distance (EMD) values were calculated as previously described (Ouellet et all, 2017).

Statistical analysis: All means are presented with their corresponding SEM. Statistical analysis was performed using GraphPad Prism 8.0.1 for MacOS (GraphPad Software Inc., San Diego, CA). Statistical significance was assessed by Student’s t test (between two groups) or one-way ANOVA with a Tukey post hoc test to identify individual differences between means. Probabilities of $P < 0.05$ were considered statistically significant.

Results

We observed a significant reduction in mitochondrial length after mitochondrial membrane potential disruption by rotenone treatment (Figure 1A). We distinguished a shift in mitochondrial length distribution between the control and rotenone treatments (Figure 1B). The EMD values (Figure 1C) and mitochondrial connectivity (Figure 1D) were both significantly decreased after rotenone treatment.

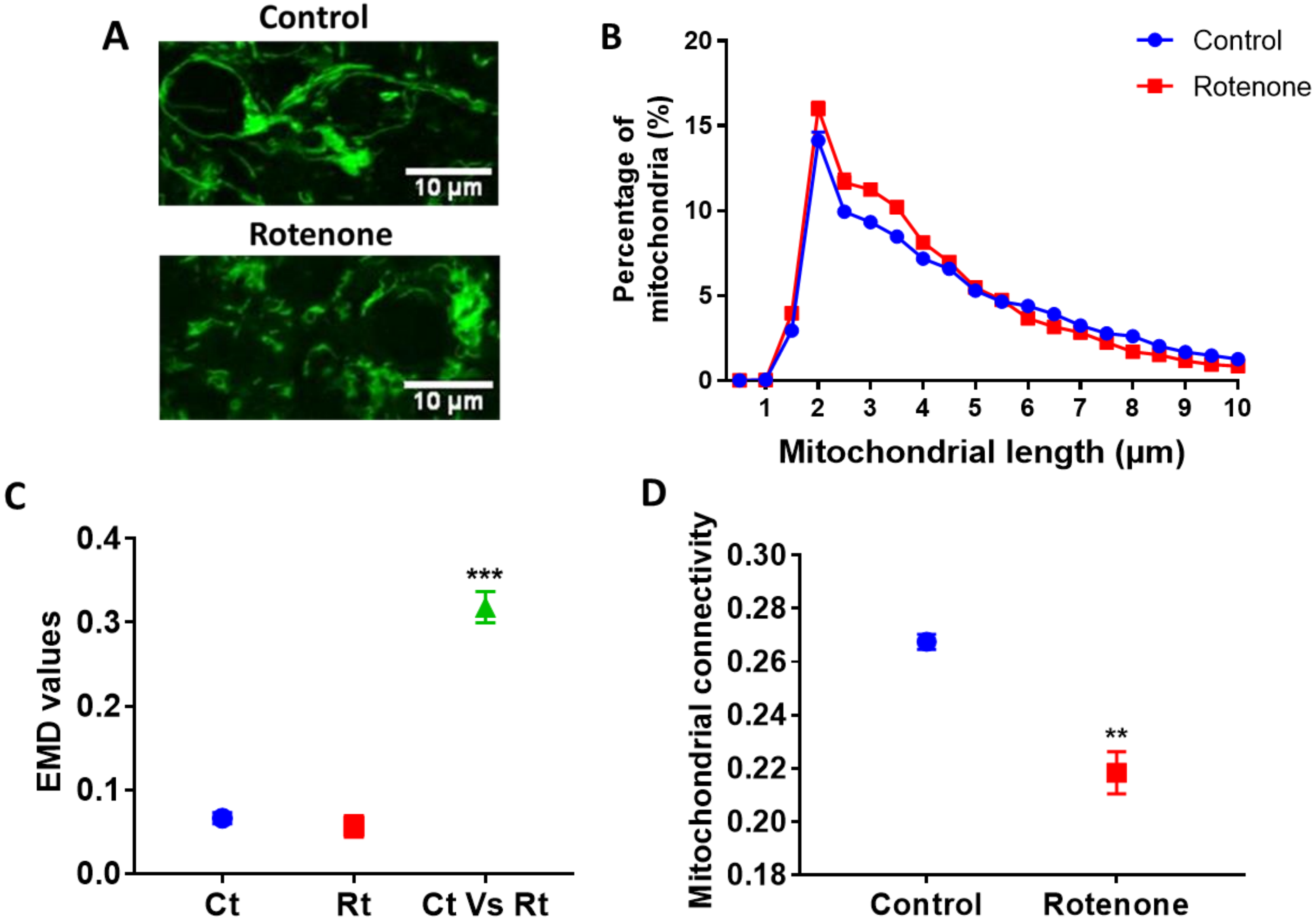

Figure 1: Momito analysis of mitochondrial morphologies stained with TMRM in cumulus cells after rotenone treatment. (A) Images of control and rotenone treated cumulus cells where a reduction in mitochondrial length was observed after treatment. (B) Quantification of mitochondrial length distribution. Data are expressed as the average of 3 independent experiments ± SEM. (C) EMD quantification of the shift in length distribution between treatment and control, ***($p < 0.001$). (D) Mitochondrial connectivity reduced by rotenone treatment, **$p<0.01$ compared to the control.

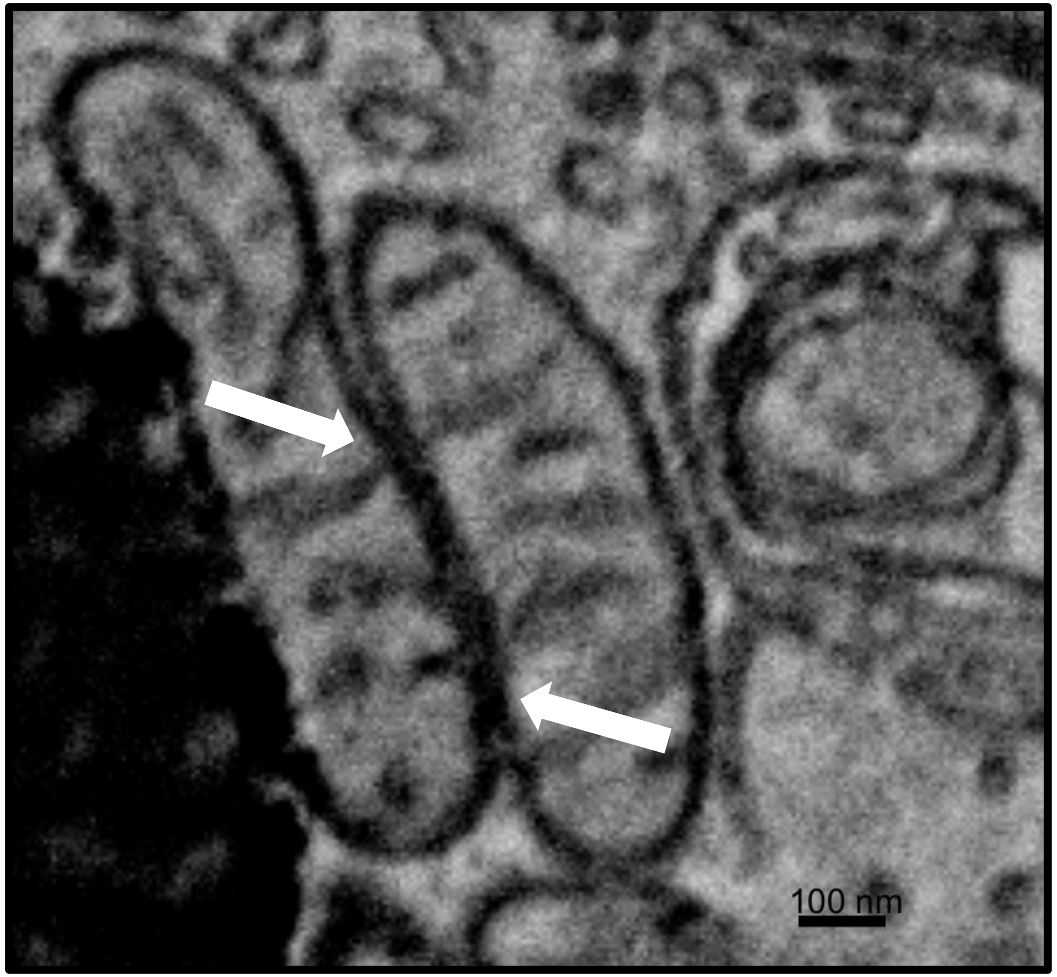

Figure 2: Inter-mitochondrial junctions with cristae alignment were observed in mitochondia from cumulus cells (white arrows).

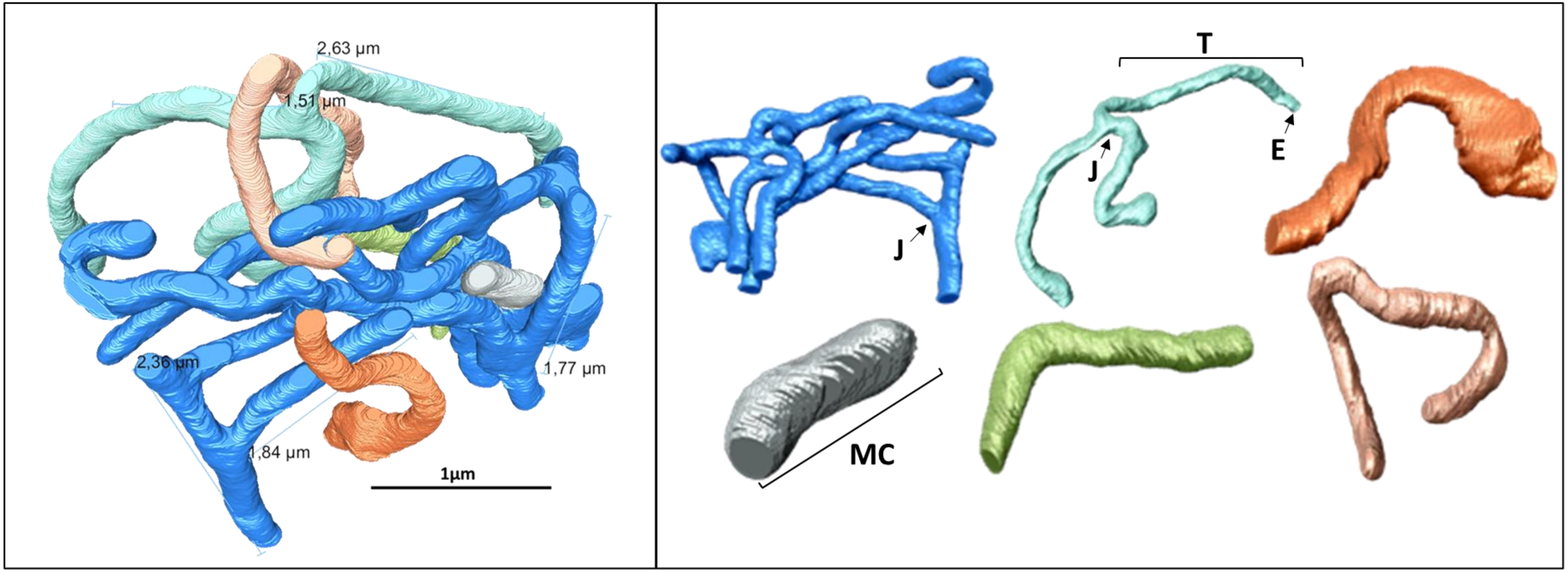

Figure 3: 3D reconstructed mitochondrial cluster containing 6 interconnected mitochondrial components **(MC)** and measuring 2,43 µm³ in volume. Several components' branches were >1 µm in length and had more than 20 elements of: ends **(E)**, tubules **(T)** and junctions **(J)**.

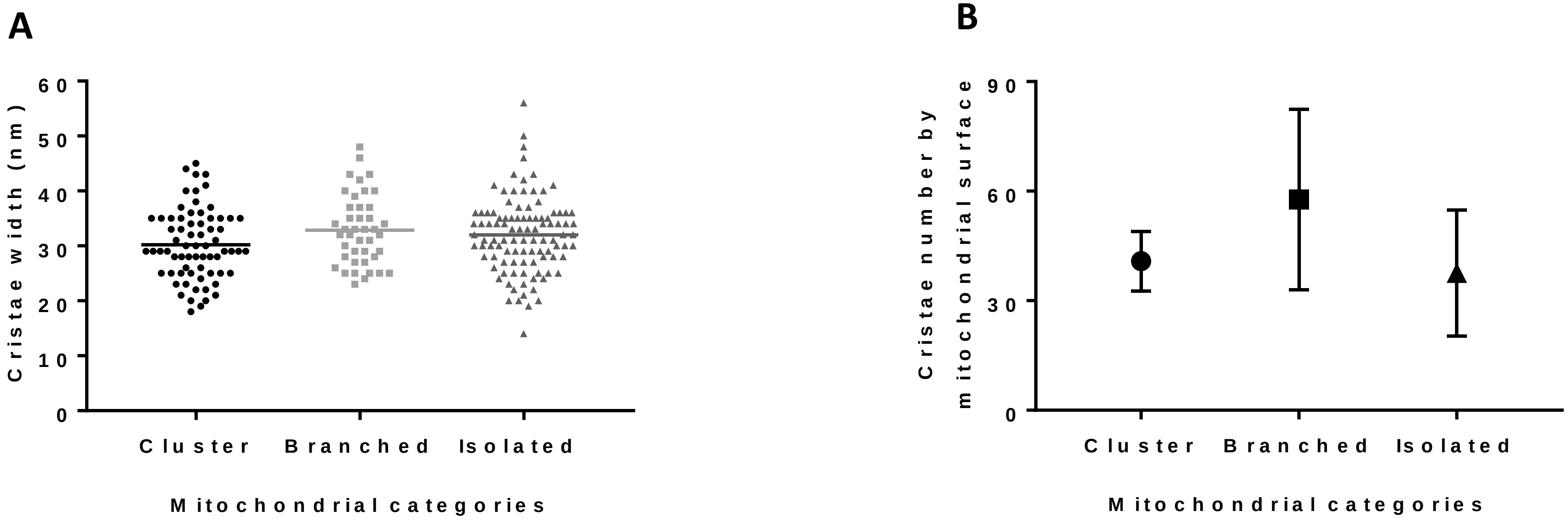

Figure 4: Cristae analysis from 3-D reconstructed mitochondrial categories. (A) cristae width. (B) ratio between cristae number and mitochondrial surface.
